# Anemia in Chronic Kidney Disease: From Pathophysiology and Current Treatments, to Future Agents

**DOI:** 10.3389/fmed.2021.642296

**Published:** 2021-03-26

**Authors:** Jose Portolés, Leyre Martín, José Jesús Broseta, Aleix Cases

**Affiliations:** ^1^Department of Nephrology, Puerta de Hierro Majadahonda University Hospital, Madrid, Spain; ^2^Anemia Working Group Spanish Society of Nephrology, Madrid, Spain; ^3^Instituto de Investigaciones Biomédicas August Pi i Sunyer (IDIBAPS), Universitat de Barcelona, Barcelona, Spain

**Keywords:** anemia, chronic kidney disease, erythropoiesis-stimulating agents, iron, HIF stabilizer, HIF prolyl-hydroxylase inhibitor, hepcidin, COVID 19

## Abstract

Anemia is a common complication in chronic kidney disease (CKD), and is associated with a reduced quality of life, and an increased morbidity and mortality. The mechanisms involved in anemia associated to CKD are diverse and complex. They include a decrease in endogenous erythropoietin (EPO) production, absolute and/or functional iron deficiency, and inflammation with increased hepcidin levels, among others. Patients are most commonly managed with oral or intravenous iron supplements and with erythropoiesis stimulating agents (ESA). However, these treatments have associated risks, and sometimes are insufficiently effective. Nonetheless, in the last years, there have been some remarkable advances in the treatment of CKD-related anemia, which have raised great expectations. On the one hand, a novel family of drugs has been developed: the hypoxia-inducible factor prolyl hydroxylase inhibitors (HIF-PHIs). These agents induce, among other effects, an increase in the production of endogenous EPO, improve iron availability and reduce hepcidin levels. Some of them have already received marketing authorization. On the other hand, recent clinical trials have elucidated important aspects of iron supplementation, which may change the treatment targets in the future. This article reviews the current knowledge of the pathophysiology CKD-related anemia, current and future therapies, the trends in patient management and the unmet goals.

## Epidemiology of Anemia of CKD

Anemia is a common complication in chronic kidney disease (CKD), and is associated with a reduced quality of life (1, 2), a worse renal survival (3), an increase in morbidity and mortality (4, 5), and higher costs (6). Several studies focused on prevalence of anemia on CKD non-dialysis dependent (NDD) report variable anemia rates up to 60%.

Anemia is more prevalent and severe as the estimated glomerular filtration rate (eGFR) declines. An analysis of the cross-sectional data from the National Health and Nutrition Examination Survey (NHANES) in 2007–2008 and 2009–2010 (7) revealed that anemia was twice as prevalent in patients with CKD as in the general population (15.4% vs. 7.6). The prevalence of anemia raised with the progression of CKD: 8.4% at stage 1 to 53.4% at stage 5. Similar data was observed in a more recent paper by the CKD Prognosis Consortium (8). In addition, they observed an increased prevalence of anemia among diabetic patients, independent of eGFR and albuminuria.

Regarding new onset of anemia, the observational study NADIR-3 followed CKD stage 3 patients without anemia during 3 years. The authors estimated an annual rate of onset of anemia of 11% in the first year, 20% in the second year and 26% in the third year. In addition, the study revealed that those that had developed anemia significantly progressed more rapidly to CKD stages 4–5, had higher rates of hospitalizations (31.4 vs. 16.1%), major cardiovascular events (16.4 vs. 7.2%) and mortality (10.3 vs. 6.6%) (9).

With regards to the “*real-world”* management of anemia in CKD, considerable controversies and variability exist in the context of guideline recommendations. However, few studies have evaluated this issue. An Italian observational study evaluated anemia management in two visits, among 755 prevalent NDD-CKD patients. Mean eGFR was 27.5 ± 10 mL/min/ 1.73 m^2^. The prevalence of severe and mild anemia was 18 and 44%, and remained unchanged at month 6 (19.3 and 43.2%). Clinical inertia to ESA was similar at baseline and at month 6 (39.6 and 34.2%, respectively, *P* = 0.487) and it was less frequent than clinical inertia to iron therapy (75.7 and 72.0%, respectively) (10). A recent observational analysis from the Swedish Renal Registry evaluated the epidemiology and treatment patterns of all nephrology-referred adult CKD patients during 2015. Among 14 415 patients [Non-Dialysis Dependent (NDD), 11,370; Dialysis-Dependent (DD), 3,045] anemia occurred in 60% of NDD and 93% of DD patients. DD patients used more erythropoiesis-stimulating agents (ESAs; 82 vs. 24%) and iron (62 vs. 21%) than NDD patients. The prescribed ESA doses were low to moderate [median 48.2 IU/kg/week (NDD), 78.6 IU/kg/week (DD)]. Among ESA-treated patients, 6–21% had hemoglobin (Hb) >13 g/dL and 2–6% had Hb <9 mg/L. Inflammation (C-reactive protein >5 mg/L) was highly prevalent and associated with ESA resistance and higher ESA doses, but not with iron use. Further, higher ESA doses (>88 IU/kg/week) were associated with an increased risk of major adverse cardiovascular events. Despite the recommendations of guidelines, the use of iron was unexpectedly low, particularly in ESA-treated NDD patients, while a fifth of the dialysis patients receiving ESA had a hemoglobin above the recommended targets (11). A multicenter cross-sectional study conducted at specialist nephrology clinics in Ireland also showed an evident variability in the implementation of different guidelines, the high rates of anemia (from 21 to 63%; *p* < 0.001, depending on the CKD Stage), the low testing rates for iron deficiency (only 45% of anemic patients), and the low use of treatment (86 % of patients with confirmed iron deficiency were not on treatment) (12).

## Physiopathology of Anemia in CKD

The mechanisms of anemia in CKD are multifactorial. The progressive reduction of endogenous erythropoietin (EPO) levels has classically been considered to play a preeminent role. However, other factors have also been described to contribute to anemia in CKD patients, such as an absolute iron deficiency due to blood losses or an impaired iron absorption, an ineffective use of iron stores due to increased hepcidin levels, systemic inflammation due to CKD and associated comorbidities, a reduced bone marrow response to EPO due to uremic toxins, a reduced red cell life span, or vitamin B12 or folic acid deficiencies (13).

### Hypoxia Inducible Factor System

EPO is a glycoprotein (30.4 kDa) that binds to its receptor on the surface of erythroid progenitor cells mainly in the bone marrow, and serves as a key stimulus for red cell survival, proliferation and differentiation. EPO is produced predominantly by the fibroblast-like interstitial peritubular cells of the kidneys, and in a much lesser proportion, by the perisinusoidal cells in the liver, in response to changes in tissue oxygen tension (14). The production of EPO is controlled at the level of the EPO gene transcription. One of the most important factors that regulate its expression is the hypoxia-inducible factor (HIF) system, whose activity depends on the tissue oxygen levels.

In more detail, under hypoxia or anemic stress, the HIF1 binds to the EPO gene, and activates its expression. HIF1 is composed of two subunits, HIF1α and HIF1β. HIF1β is constitutively expressed whereas HIF1α is virtually absent under normoxic conditions. However, in low oxygen tension settings, HIF1α accumulates and translocates to the nucleus, where it binds to HIF1 β. The HIF1 α-β heterodimer binds to DNA sequences called hypoxia response elements (HRE), regulating the expression of various hypoxia-sensitive genes, either downregulating or upregulating them. The purpose of this rapid adaptive response is to protect against cellular damage, by improving oxygen delivery and by decreasing oxygen consumption. Among these hypoxia-sensitive genes is the EPO gene, which is activated, leading to an increased EPO production. Other genes that are transcriptionally upregulated by the HIF complex are those encoding EPO receptor, transferrin and transferrin receptor, vascular endothelial growth factor (VEGF) or endothelin-1 (15). Recent work has shown that the HIF transcription factors are key elements in the control of cell metabolism and function (16–18). An effect of HIF on total and LDL-cholesterol levels has also been described (19), probably in part by the effects of HIF on degradation of the rate-limiting enzyme, 3-hydroxy-3-methylglutaryl-CoA reductase (20), similar to what has been observed in high altitude settings (21).

**Figure 1:**
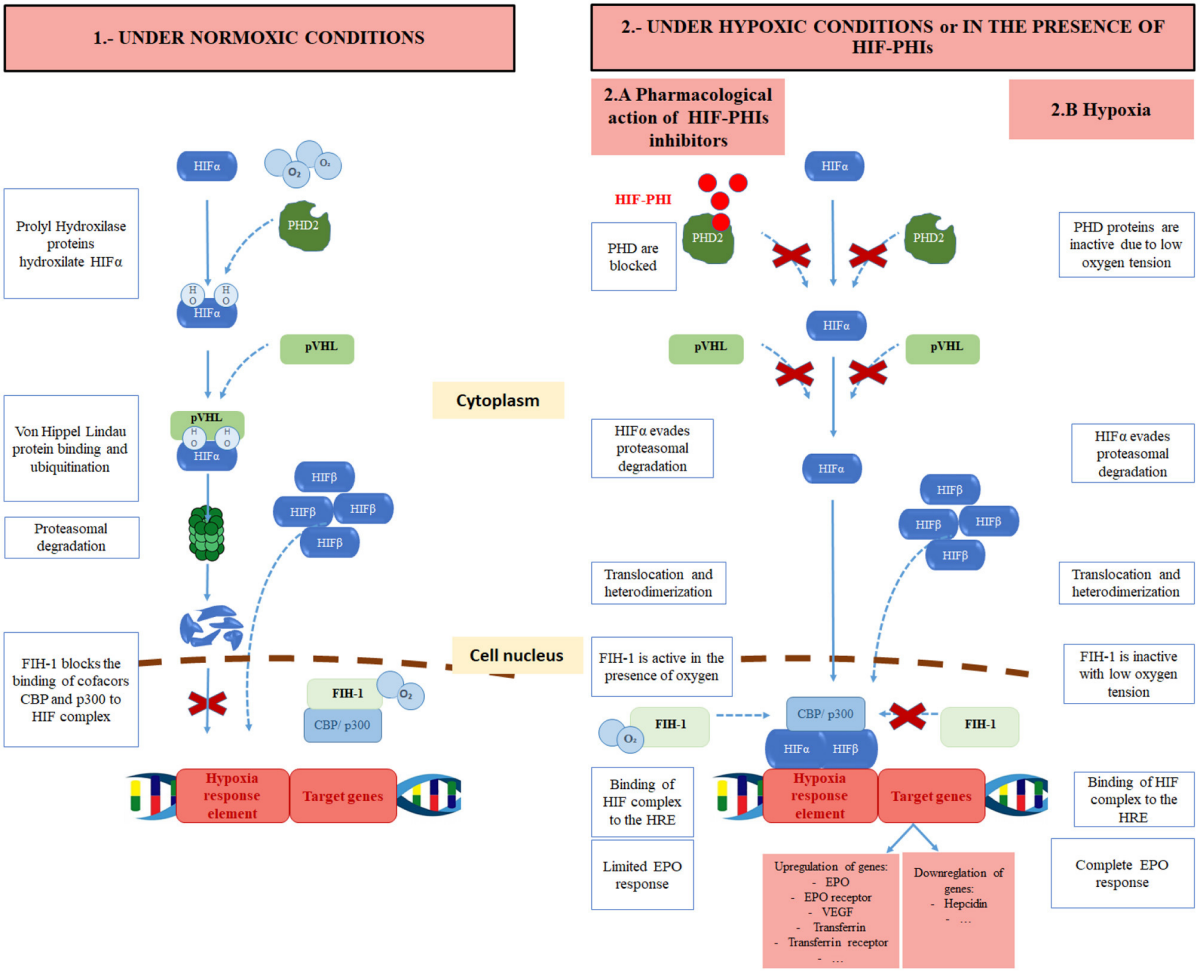
Regulation of HIF under normoxic conditions, pharmacological effects of hypoxia-inducible factor prolyl hydroxylase inhibitors (HIF-PHIs) and under hypoxic conditions. HIF, hypoxia-inducible factor; O_2_, oxygen; OH, hydroxyl; PHD, prolyl hydroxylase domain protein; HIF-PHI, hypoxia inducible factor prolyl hydroxylase inhibitor; pVHL, Von Hippel Lindau protein; FIH-1, Factor inhibiting HIF; CBP, CREB-binding protein.

Under normoxic conditions, HIF1α is degraded. For this purpose, HIF1α is hydroxylated at two proline residues. This hydroxylation is performed by specific HIF prolyl-hydroxylase enzymes called prolyl hydroxylase domain (PHD) enzymes that need the presence of oxygen, iron, and 2-oxoglutarate as co-factors. Three forms have been described: PHD1, PHD2, PHD3. PHD2 is the main isoform regulating HIF activity (22). Once HIF1α is hydroxylated, the E3 ubiquitin ligase von Hippel-Lindau (pVHL) binds HIF1α, and is targeted for proteasomal degradation. In contrast, under low oxygen tension the action of PHDs is prevented, allowing for HIF1α stabilization and translocation to the nucleus (23, 24). This pathway is the target of the new so-called hypoxia-inducible factor prolyl hydroxylase inhibitors (HIF-PHIs) (Figure 1).

HIF activity is also regulated through the hydroxylation at a carboxy-terminal asparagine residue of HIF1α by factor-inhibiting HIF (FIH) (25). This hydroxylation of HIF1α occurs with normal oxygen levels and reduces its transcriptional activity. Indeed, it prevents HIF from recruiting transcriptional coactivators such as p300 or CBP, that are needed for the transactivation of hypoxia-responsive genes (26, 27). Furthermore, angiotensin II, which is often found to be increased in CKD patients, raises the production of reactive oxygen species, leading to an inhibition of PHD enzymes, and therefore, a rise in EPO levels (28–30).

Nonetheless, recent animal studies have described important roles to other components of the HIF system. Three isoforms of HIFα have been described, that share similarities regarding oxygen-dependent hydroxylation: HIF1α, HIF2α, and HIF3α. All of them can bind to the HIFβ subunit. However, there may be some differences amongst them: while HIF1α and HIF2α activate gene transcription, HIF3α downregulates HIF1α and HIF2α activity. Moreover, their effect on the expression of some genes may also vary. HIF2α may play a more important role than HIF1α in the regulation of EPO production, as it is specifically required for renal and hepatic production of EPO. The direct role of HIF3α on erythropoiesis has not been fully described. Finally, the expression of HIF2α and HIF3α is limited to several tissues, while HIF1α is ubiquitous (31).

### EPO Production in CKD

In CKD patients, EPO levels are inadequately low with respect to the degree of anemia. EPO deficiency starts early in the course of CKD, but it appears that when eGFR falls below 30 ml/min per 1.73 m^2^ this deficiency becomes more severe (32). This absolute EPO deficiency can be caused by a decrease in the EPO production and/or by errors in EPO-sensing. CKD associates an alteration in oxygen delivery to the kidneys due to a reduced blood flow. This results in an adaptation of renal tissue to consume less oxygen and the subsequent maintenance of a normal tissue oxygen gradient. As a consequence, PHD enzymes remain active, the HIF heterodimer is not formed and the EPO gene is not activated (33).

Furthermore, it has been demonstrated experimentally that hypoxia-induced EPO production is inhibited by some inflammatory cytokines such as interleukin-la (IL-la), IL-l beta, transforming growth factor-beta (TGF-beta), and tumor necrosis factor-a (TNF-a) (34, 35). It is well-known that CKD itself leads to an increase of inflammation and immune activation molecules, which would inhibit hypoxia-induced EPO production (36, 37). However, this mechanism of EPO production seems to be blunted rather than abolished in some CKD patients, as they are able to produce additional endogenous EPO in their kidneys and liver under certain circumstances. For instance, when exposed to high altitude or bleeding. Apparently, augmentation of HIF signaling can revert quiescent EPO-producing and oxygen-sensing (REPOS) cells back to EPO production (38). This has been confirmed in observational studies, where hemodialysis patients living in higher altitude require lower doses of recombinant human EPO (rhuEPO) (39).

Some CKD patients may also present with a functional EPO deficiency or EPO resistance, where normal range EPO levels coexist with low hemoglobin (Hb) levels (40), indicating that the bone marrow response to endogenous and exogenous EPO is blunted in patients with CKD. The mechanisms that have been hypothesized for the EPO resistance are various: Proinflammatory cytokines are thought to induce apoptosis as well as a to have a direct toxic effect *via* the induction of labile free radical nitric oxide on erythroid progenitor cells (41, 42). Proinflammatory cytokines are thought to downregulate the expression of EPO receptor on their surface too. It has also been shown that cytokines can induce the production of antagonistic peptides that bind to the EPO receptor, and inhibit the EPO-dependent proliferation (43–46). Moreover, hepcidin (whose production is enhanced by inflammation) might also contribute to EPO resistance, by directly inhibiting erythroid progenitor proliferation and survival (47). Lastly, neocytolysis is a homeostatic physiological process that leads to selective hemolysis of young circulating red blood cells, that has been found to contribute to resistance in CKD patients receiving exogenous EPO (48).

**Figure 2:**
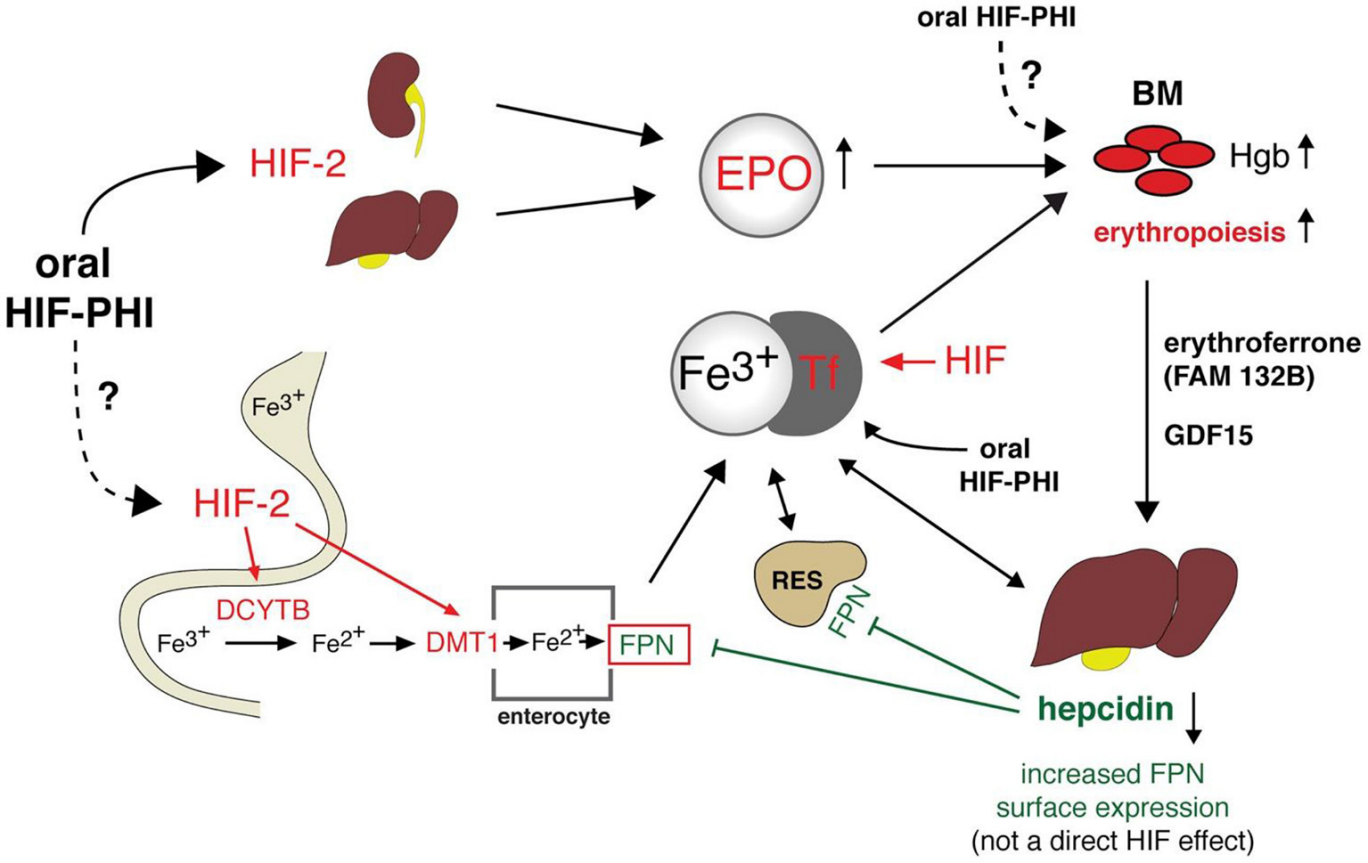
Integrated model for CKD-anemia physiology and actual and potential treatments. HIF, hypoxia-inducible factor; Fe, iron; HIF-PHI, hypoxia inducible factor prolyl hydroxylase inhibitor; EPO, Erytrhopoietin; Tf, transferrin; DCYTB, duodenal cytochromeb; DMT1, divalent metal transporter 1; BM, Bone marrow; FPN, ferroportin; GDF15, Growth differentiation factor 15; Hgb, hemoglobin; RES, Reticuloendothelial system; FAM132B, Gene that codes for erythroferrone.

The search for new therapeutic options for these patients is essential. In this sense, some molecules of the HIF system have already been studied as new targets for anemia treatment with a view to increase endogenous EPO production and presumably improve the utilization of iron stores (Figure 2).

### Iron Metabolism

Iron is required for an adequate erythropoietic response to EPO, and in anemic conditions having iron deficiency corrected allows lower exogenous EPO supplies (49).

Furthermore, iron is required in other essential non-erythropoietic effects that may help explain symptoms such as impaired exercise performance, cognitive impairment or decreased quality of life, and an increased risk in hospitalization or death in patients with heart failure and reduced ejection fraction, independent of anemia. Thus, these advocate for the need to correct iron deficiencies independent of Hb status (50). Iron is an essential component of myoglobin, which transports oxygen in the muscle cells. Iron also plays a substantive role in several oxidative reactions affecting intracellular metabolism, such as the electron transport chain or oxidative phosphorylation. Iron is involved in different mechanisms of DNA synthesis, degradation and repair. Finally, iron is an important component of cytochrome P450 family (51). In fact, there is an increasing evidence from observational studies that iron deficiency is associated with worse outcomes in CKD patients (52–54).

Most of the iron requirements are provided by recycling the iron present in senescent erythrocytes and the release of iron from storage sites (Figure 2). The proportion of iron that comes from the dietary uptake is much smaller. In addition, there is no physiological mechanism to regulate iron excretion. It is lost from the desquamation of intestinal epithelial cells, skin cells and blood loses and dietary iron absorption, which is regulated by hepcidin, compensate these loses.

The iron content of macrophages from the phagocytosis of senescent red blood cells, hepatocytes or enterocytes (dietary iron absorbed in the duodenum) is released into the circulation by ferroportin, the only iron exporter known. Iron is then transported through the circulation by transferrin, and delivered into target cells by transferrin binding to transferrin receptor (55). Transferrin receptors are regulated by intracellular iron quantity and cell growth. A circulating acute-phase protein produced in the liver, hepcidin, is the key regulator of iron metabolism. Its purpose is to maintain adequate systemic iron levels. Hepcidin is thought to decrease the absorption of iron in the doudenum by downregulating the expression of apical divalent metal transporter 1 (DMT1) in the enterocyte. Besides absorption, hepcidin also plays a role in iron storage. Indeed, hepcidin promotes the internalization of ferroportin into the cell for its degradation, and thus preventing iron form exiting into the circulation from enterocytes, macrophages or other iron stores. Iron overload increases hepcidin levels, whereas iron deficiency reduces its concentrations (55).

Absolute and relative iron deficiency are frequent conditions in CKD patients. Blood losses, for instance, due to blood left in the hemodialysis circuit are common. In addition, the “uremic” state and other comorbidities causing inflammation prevent from an adequate intestinal iron absorption and from the release of iron from the body stores. Proinflammatory cytokines contribute to a functional iron deficiency in several ways: They stimulate the hepatic synthesis of hepcidin, they induce the expression of DMT1 in macrophages, and induce the expression of ferritin, and inhibit that of ferroportin. They also promote the uptake of iron bound to transferrin into macrophages, *via* the transferrin receptor (56). Moreover, hepcidin is eliminated by kidney and its clearance is reduced as eGFR declines. All these mechanisms favor intracellular iron storage, limiting the availability of iron in CKD.

Under stress erythropoiesis, EPO suppresses hepcidin synthesis *via* erythroferrone (ERFE). ERFE is a hormone produced by erythroblasts in response to EPO (57). Also, HIF 1α and probably HIF2α regulates hepcidin production by directly binding to and repressing its promoter (58), while HIF-2α enhances iron availability through the activation of genes encoding DMT1 and duodenal cytochrome b (DCYTB) required for transport of dietary iron from the intestinal lumen and for the import of lysosomal iron arising from the circulation (59).

## Current Clinical Practice Guidelines of Anemia in CKD

Anemia management in CKD has evolved dramatically: from the first oral iron supplements introduced in 1830s (ferrous sulfate), the use of red blood cell transfusions along the XX century, the appearance of the first rhuEPO use in late 1980s followed by long-acting ESAs, to finally, the widespread use of intravenous iron supplements in recent years. However, the actual management of anemia in patients with CKD varies among different countries and medical units (60, 61). Indeed, current guidelines KDIGO (13)—ERBP (62)—NICE (63) do not fully coincide with each other. Some controversies exist about the optimal Hb and iron targets. Table 1 summarizes the main differences.

**Table 1:** Summary of the key recommendations of the most recent anemia guidelines.

	**Diagnosis of iron deficiency**	**Treatment initiation**	**Hb target under treatment with ESAs**	**SF and TSAT objectives in patients under treatment**	**FE oral vs. IV**
NICE (2015)	**Test every 3 months (1–3 m in HD)** - Use %HRC > 6%, only if blood processing within 6 h. - if not possible, use CHr < 29 pg - If not, use a combi-nation SF < 100 ng/mL and TSAT < 20%	**Correct iron deficiency before ESA therapy**. - Patient-centered: discuss risks benefits of treatment options. Take into account the person's choice. Avoid Hb < 10 g/dL.	**Hb 10–12 g/dl**	**Avoid SF >** **800 ng/mL** To prevent this, review iron dose if SF > 500 ng/mL	**ND-CKD with anemia and iron deficiency:** - offer a 3 months trial of oral iron therapy. - If it fails, offer IV iron therapy. **- DD-CKD:** Preference for IV iron - **If IV iron, consider high dose, low frequency** formulations for ND and DD-CKD patients.
KDIGO (2012)	**SF** **≤** **100 ng/mL and TSAT** **≤** **20%**.	**A trial with IV iron** if Hb increase or ESA dose reduction is desired and SF ≤ 500 ng/mL and TSAT ≤ 30% **ND-CKD: When Hb** **<** **10 g/dL:** Individualize decision based on the rate of fall of Hb, risks and symptoms. **DD-CKD:** When Hb 9-10 g/dL. Avoid Hb < 9 g/dl.	**Hb** **≤** **11.5 g/dl** - **Target to Hb** **>** **11.5 g/dl if QoL improve** is foreseen and patient accepts risks. Avoid Hb >13 g/dL	**Stop iron supplements** if SF > 500 ng/mL	**ND-CKD: Select route based on severity of ID, prior response, side effects, costs**, A trial of iv iron, or a 1–3 month trial of oral iron therapy. **- DD- CKD**: Preference for IV iron
ERBP (2009)	**SF** **<** **100 ng/mL and TSAT** ** < 20% if ESA naïve**. **SF** **≤** **300 ng/mL and TSAT** **≤** **30% if ESA treated**	**Avoid Hb** **<** **10 g/dL**. - If low risk patients or a benefit in QoL foreseen ESA could start at ↑ Hb (avoid Hb >12 g/dL) - In high risk patients with worsening heart disease, treatment initiation at Hb9-10 g/dL.	**Hb 10–12 g/dl** - High risk patients with asymptomatic disease: target Hb around 10 g/dL	**Avoid SF** **>** **500 ng/ml and TSAT** **>** **30%**.	**ND-CKD and mild-moderate anemia:** Oral iron as first line therapy for > 3 months. **ND-CKD and severe anemia or when oral iron ineffective:** IV iron as first choice.

*SF, serum ferritin; TSAT, Transferrin saturation; %HRC, percentage of hypochromic red blood cells; CHr, hemoglobin content in reticulocytes; Hob, Hemoglobin; ND-CKD, Non dialysis dependent Chronic kidney disease; DD-CKD, dialysis dependent CKD; QoL, quality of life; IV, intravenous ESA erythropoiesis stimulating agent; Fe, iron*.

Further, these guidelines do not include more recent studies assessing the efficacy and safety of IV iron, as well as different strategies of iron repletion, which will probably change the clinical practice in the future. They demonstrate that in ND-CKD, renal transplant and PD patients IV iron is more efficacious and safe. In addition, a high-dose low frequency administration strategy in dialysis dependent chronic kidney disease (DD-CKD) patents is safe and improves outcomes in patients (63).

## CKD Anemia Treatment

### Erythropoiesis-Stimulating Agents (ESAs)

The first EPO analog available was epoetin α and short time later epoetin β. It is produced by recombinant DNA technology in cell cultures. Darbepoetin alfa (DA) and methoxy polyethylene glycol-epoetin beta where developed thereafter and presented a prolonged half-life. More recently, biosimilars of the original epoetin have been introduced in the market.

Not all ESAs are equal. They have different pharmacokinetic and pharmacodynamic properties, such as different half-lives and EPO receptor affinity, allowing a less frequent dosing and ease of administration for NDD CKD patients with long-acting ESAs. In addition, it is important to point out the fact that the conversion factor between short-acting and long-acting ESAs is likely not linear. In fact, at higher doses, long-acting ESAs are more dose-effective (64). However, based on efficacy and safety data, various Cochrane metaanalysis advocate for insufficient evidence to suggest the superiority of any ESA formulation or any ESA administration pattern (65, 66).

Some observational studies have shown conflicting results regarding such outcomes. For instance, the Study of the Japanese Registry of Dialysis that showed a 20% higher risk of mortality from any cause in patients treated with long-acting ESAs compared to those treated with short-acting ESAs (67). On the contrary, an Italian observational study in ND-CKD that showed a higher risk of progression to ESKD and mortality in patients receiving short-acting ESAs at high doses (68). These results should be taken cautiously due to the study design and the risk of bias. In contrast, a recent randomized controlled trial (RCT) comparing monthly administration of CERA with reference shorter-acting agents epoetin alfa/beta and DA, showed non-inferiority regarding Hb target achievement, major adverse cardiovascular events or all-cause mortality in NDD and DD-CKD. It was however observed that patients who did not achieve levels of Hb above 10 g/dL or those at the highest quartile of ESA dose, had a higher risk of CV events or death, independent of the assigned treatment (69). More RCTs are needed to assess the differences between different ESA formulations and administration patterns, particularly in patients requiring higher doses of ESA.

### Individualizing Hb Target According to the Patient Profile

The target Hb concentration during ESA therapy is still controversial. Studies early after the appearance of rhuEPO demonstrated its efficacy in reducing the need for blood transfusions, the symptoms related to anemia and an improved quality of life (70, 71). Various landmark trials have dwelt on the convenience of a complete correction of anemia. Indeed, in the CHOIR trial the use of a target Hb level of 13,5 g/dl was associated with increased risk of suffering a composite of death, myocardial infarction, hospitalization for congestive heart failure and stroke, and no incremental improvement in the quality of life. CREATE trial did not observe an increase of the risk of cardiovascular events, but showed an increase in the necessity of dialysis among the group that targeted Hb in normal range (13–15 g/dl) The TREAT trial compared the use of darbepoetin alfa targeting Hb level of 13 g/dl vs. a rescue therapy when Hb level dropped below 9 g/dl in patients with diabetes, NDD-CKD and moderate anemia. The use of darbepoetin alfa did not reduce the risk of either of the two primary composite outcomes (either death or a cardiovascular event or death or a renal event), and was associated with an increased risk of stroke (65, 72–75). Yet, it is still unclear whether this increased risk is due to the higher ESA doses and the possible non-erythropoietic effects, whether it is due to the underlying systemic inflammation in patients with ESA-hyporresponsiveness, rather than due to the high Hb level itself (76).

Conversely, although some trials have demonstrated significant improvements in quality of life (QoL) in patients targeted to normal Hb levels (73, 75, 77) the clinical relevance of these findings are questioned. The results of these trials were highly influential in changing the guidelines and clinical practice of anemia in NDD-CKD.

Current evidence, then, demonstrates a clear benefit in correcting Hb levels if they are below 10 g/dL, but also an increased risk when exceeding Hb 13 g/dl. The Hb target, then, appears to be somewhere in between 10 and 12 g/dl. Individualizing the Hb target relative to the patient's risks, basal conditions and preferences is advisable.

### Iron Supplementation for Anemia in CKD

Guidelines acknowledge that the optimal strategy to manage iron metabolism remains unclear, and advocate for balancing the potential benefits and risks of iron supplementation (13, 62, 63). Table 1 summarizes the principles and targets of the management of iron supplements of the KDIGO (13)—ERBP (62)—NICE (63) guidelines. In recent years some good quality pre-clinical studies, clinical trials and epidemiological studies have shed some light on the therapeutic approach regarding iron deficiency in CKD and will surely change clinical practice.

Intravenous (IV) iron has shown benefits both in DD-CKD and more recently in NDD-CKD, as it has proved to be more efficacious in rising ferritin and Hb levels, while reducing ESA and transfusion requirements. Specifically, in hemodialysis patients, oral preparations seem to be useless, maybe except for the phosphate binder ferric citrate (78). In addition, gastrointestinal intolerance and constipation reduce tolerance and compliance of oral iron formulations (79).

However, some concerns raised about IV iron formulation such as enhanced oxidative stress, endothelial dysfunction or the potential role in favoring infection. Further, IV iron administration has been associated with an increased risk of hypotension, headaches or hypersensitivity reactions. Labile iron, which is the iron that is freed into the circulation after administration and non-bound to transferrin, is an important cause of such adverse reactions.

IV iron supplements are non-biologic complex drugs. An iron core, covered by a complex structure of polysaccharides forms them. Indeed, the differences in the structure of the molecule among different IV iron formulations may be responsible for the differences in outcomes of each IV iron formulation. Some studies have even demonstrated differences in the attainment of Hb levels between the “original” brand and its generic form of iron sucrose (80).

On the other hand, there is growing evidence that oral compound can have a deleterious effect on gut microbiota which may worsen uremic dysbiosis (81, 82). Whether oral iron induced changes in gut microbiota, increases uremic toxins production and/or inflammation in CKD remain to be elucidated.

### Dosing Patterns: The “Iron First-Approach” and the “High Dose-Low Frequency Approach”

As mentioned before, iron is essential for an adequate erythropoesis. In this sense, several trials have demonstrated that the correction of iron deficiency lessens the need for ESA in CKD patients (FIND:CKD) (49). Hence, results from TREAT study demonstrate that control group receiving only IV iron but no ESAs may increase Hb by 1 g/dl. The so called “iron fist approach” suggested by guidelines (CITA) is based in his efficacy for anemia correction. Unfortunately, we have no evidence of the effect on hard end-points. Moreover, the risk of Hb overshooting depends on high levels of EPO but no IV iron use, since iron is not a growth factor.

In addition, various studies carried out in patients with heart failure (HF) with reduced ejection fraction and iron deficiency demonstrated that IV iron supplementation [but not oral iron, (83)] have shown an improvement outcomes, HF symptoms, functional class and quality of life, (FAIR-HF, CONFIRM-HF, EFFECT-HF) (84–86). Further, a meta-analysis demonstrated that these benefits of IV iron therapy were independent of the presence of anemia. More recently, a reduction of hospitalizations for HF has also been demonstrated in a study among patients with acute heart failure and iron deficiency (AFFIRM_AHF) (87). Likewise, an improvement in renal function has been observed with IV ferric carboximaltose in a subanalysis of the FAIR-HF study (88). In a small study in patients with HF, CKD and anemia with iron deficiency, IV iron was associated with an improvement of myocardial function and of cardiac dimensions (89), similar to the observations in another pilot study (90). There is a considerable correlation between heart failure, iron deficiency and renal failure and each comorbidity reduces the survival of these patients (91). Large registry data show that CKD is present in 12 to 74% of HF cases (92) and that its prevalence increases as renal function declines.

The Anemia Working Group of the Spanish Society of Nephrology published a review advocating for this “iron first-approach” and recommending the administration of IV ferric carboxymaltose in patients with CKD, HF with reduced ejection fraction and iron deficiency, even in the absence of anemia, extrapolating the recommendations of the Heart Failure Guidelines of the European Society of Cardiology (93, 94).

Moreover, newer IV iron formulations are more stable and have safer profiles that allows the administration of higher doses of iron per session (95, 96). The recent PIVOTAL trial has confirmed the efficacy and safety of high-dose IV iron sucrose: it is a UK open label, randomized controlled trial among 2,141 incident hemodialysis patients, that compared a proactively administered high-dose IV iron regimen with a reactively administered low-dose regimen. The trial demonstrated that a proactive high-dose schema reduced the death of all causes or an aggregated of non-fatal cardiovascular events [HR de 0.85: IC 95% (0.73–1.0); *p* < 0.001 for non-inferiority and *p* = 0.04 for superiority]. In addition, the high-dose regimen was not associated with higher risks of death, major adverse cardiovascular events, or infection (97). These findings should lead to a change in clinical guidelines.

### Iron Targets: How Much Iron Is Too Much Iron?

Iron overload is a condition of elevated body iron content associated with signs of organ dysfunction that is presumably caused by excess iron. Some studies have demonstrated an increase in the liver iron content in hemodialysis patients, and an association between hepatic iron overload and hepatic steatosis has been recently described (98). However, its clinical relevance is still not known, and no deposits have been observed in other territories, such as cardiac or pancreatic (99–101). A metaanalysis of clinical trials and observational studies in the setting of hemodialysis suggests that patients that received higher doses of IV iron did not show a higher risk of mortality, infections or cardiovascular events (102). Nonetheless, the strength of the findings is limited by the small number of patients and of events in the clinical trials, and by the statistical heterogeneity in the observational studies included.

A recent epidemiological study has shown a slight higher mortality risk in patients with NDD-CKD and ferritin levels above 500 ng/ml, compared to patients with no iron deficiency, and patients with absolute or relative iron deficiency (40). These findings should be taken cautiously due to the presence of possible confounding factors. On the contrary, incident hemodialysis patients in the proactive high dose iron regimen in the PIVOTAL study showed a reduced risk in the primary end point (composite of death, MI, stroke, hospitalization and HF), as mentioned above in this article, and achieved higher mean ferritin levels (without exceeding 700 ng/ml, as per protocol). The upper limits of iron targets and the long-term safety of high doses of IV iron supplementation, specially of the accumulated high iron doses in hemodialysis patients, still needs to be clarified.

There has long been a concern whether iron supplements increased the risk of infections. A sub-analysis of PIVOTAL study did not show differences in infection episodes, hospitalization or death for infection between the proactive high dose regimen and reactive low dose-iron groups of patients (103).

### Hypoxia-Inducible Factor Prolyl Hydroxylase Inhibitors

In recent years, new drugs have been developed for the treatment of anemia. These are the so called HIF-prolyl-hydroxylase inhibitors (HIF-PHIs). These drugs inhibit the action of prolyl-hydroxylase, which leads to an increase in the levels of HIF, and therefore, to an increase in endogenous EPO (24). There are currently four HIF-PHIs undergoing phase III clinical trials: roxadustat, daprodustat, vadadustat and molidustat. All of them are administered orally. However, they have differences in pharmacodynamics and pharmacokinetics, which probably determine differences in their interaction with the HIF system, and thus lead to differences in efficacy and safety profiles (104) (Table 2).

**Table 2:** Pharmacological characteristics and current knowledge status of different Hypoxia-inducible factor prolyl hydroxylase inhibitors.

	**Roxadustat (FBG-4592)**	**Vadadustat (AKB-6548)**	**Daprodustat (GSK 1278863)**	**Molidustat (BAY-3934)**
Affinity IC50 (uM)	0.027	0.029	0.067	0.007
PHD Isoform Selectivity	PHD 1-3	PHD 3>2	PHD 2-3	PHD 2 < 1 and 3
HIFα selectivity	HIF1α and 2α	HIF2α > 1α	HIF1α and 2α	HIF1α and 2α
Inhibitory concen-tration IC 50 (μM)	>100	29	21	65
Half life humans (h)	12	4.5	4	Not available
Current status of development	2019 approval in Japan and China Phase III reported at ASN 2019	Ongoing phase III Preliminary report at ASN 2020	Ongoing phase III	Completed phase II
Phase III clinical trials	DD (HD/DP) and NDD Correction and maintenance	DD (HD/DP) and NDD Correction and maintenance	DD (HD/DP) and NDD Correction and maintenance	Not available

*IC50, half maximal receptor inhibitory concentration; PHD, prolyl hydroxylase domain protein; HIF, hypoxia-inducible factor; ASN, American Society of Nephrology; DD, Dialysis Dependent; HD, Hemodialysis; DP, Peritoneal dialysis; NDD, Non-dialysis dependent*.

Other compounds such as desidustat (Zyan1; Cadila Healthcare), JNJ-42905343 (Janssen) or enarodustat (JTZ-951; Japan Tobacco/Akros Pharma), have completed phase II studies or are in early stages of development.

### Efficacy of HIF Prolyl Hydroxylase Inhibitors

Roxadustat is the most advanced HIF-PHI under clinical development, which has already been approved in China and Japan. Two phase 3 studies were published in 2019 comparing roxadustat with placebo in NDD, and with epoetin alfa in DD-CKD patients in China. These studies had a relative small sample size a study population and of short duration. The former compared roxadustat with placebo, without adjuvant iron supplements, and demonstrated its efficacy in rising hemoglobin levels after 9 weeks (105). The latter compared roxadustat with epoetin alfa, with iron supplement only as a rescue therapy. After 26 weeks of follow up, the attained hemoglobin levels in the roxadustat group were non-inferior to those in the epoetin alfa-arm, and both groups had a similar safety profile (106). These results were similar to those found by a phase 3 study comparing roxadustat to ESAs in hemodialysis and peritoneal dialysis patients in Japan (107, 108).

The results of several phase III clinical trials were presented in the past 2019 and 2020 Annual Meetings of the American Association of Nephrology. The ROCKIES, PYRENES and SIERRAS studies compared roxadustat vs. epoetin alpha in prevalent HD patients (109–111). The HIMALAYAS study compared roxadustat vs. epoetin alpha in incident HD patients (112). In a pooled analysis (*n* = 3.917) roxadustat was significantly superior to EPO in anemia correction and the roxadustat group received fewer transfusions 9.5 vs. 12.8%; HR (95% CI) =0.82 (0.689, 0.99). In prevalent DD patients the risk of major cardiovascular events (MACE) was comparable between the two treatment arms, whereas there was a 16% reduction in the risk of MACE plus [HR = 0.84 (0.73, 0.97); *p* = 0.02] in the roxadustat group. (MACE+: Mace plus heart failure and thromboembolic events). Interestingly, patients receiving roxadustat had reduced iron needs, and those on roxadustat and an elevated C-reactive protein were able to increase Hb levels. Among 1.526 incident DD patients, there was a 30% reduction in the risk of MACE and a 34% reduction in the risk of MACE+: [HR (95% CI) = 0.70 (0.51, 0.97); *p* = 0.03] and [HR 0.66 (0.50, 0.89); *p* = 0.005] respectively, among patients receiving roxadustat (113).

The OLYMPUS, ALPS, and ANDES trials evaluated roxadustat vs. placebo in NDD-CKD patients (110, 114, 115). An integrated analysis (*n* = 4.270) showed that roxadustat was efficacious in achieving and maintaining Hb levels, with lower risk of rescue therapy. Regarding adverse events, both arms of treatment had comparable safety profiles regarding cardiovascular events and all-cause mortality (113).

The results of the DOLOMITES trial were presented i in the past ERA-EDTA congress in June 2020 (116) and in the 2020 ASN Annual Meeting (117). This phase 3, randomized, open-label, active-controlled study evaluated the efficacy and safety of roxadustat compared to DA in the treatment of anemia in NDD- CKD patients. The median time of follow up was 104 weeks and the study enrolled 616 adult anemic patients with CKD stages 3–5. Roxadustat was non-inferior to DA in the primary endpoint, which was the achievement of Hb response during the first 24 weeks of treatment. Regarding secondary efficacy endpoints, roxadustat was superior in decreasing low-density lipoprotein cholesterol and in time to first IV iron use. Roxadustat was non-inferior in blood pressure control and time to first occurrence of hypertension, in changes in Quality of life scores, and in Hb change. The occurrence of treatment-emergent adverse events (TEAEs) was similar between the two groups, and the TEAEs leading to withdrawal of treatment were more frequent in the roxadustat group. They reported no significant differences between groups in adjudicated cardiovascular events. In all the Roxadustat studies the roxadustat patients presented an early and sustained LDL-reduction as a pleiotropic effect.

Another compound, vadadustat has also been approved in Japan in 2020. The results of two phase III trials comparing vadadustat vs. DA in Japanese HD and non-dialysis dependent patients were presented in past 2019 ASN annual meeting (NCT03439137 and NCT03329196, respectively). Over 300 patients were followed for 52 weeks in each study. In both trials, vadadustat showed non-inferiority in maintaining Hb levels within the target range and a similar safety profile. Additionally, vadadustat was associated with an increase in total iron binding capacity and a decrease in hepcidin in DD and NDD-CKD (118, 119). Furthermore, the results of the PRO2TECT study (NCT02648347) were presented at the 2020 ASN annual meeting. It consists of two randomized, phase 3, global, open-label, sponsor-blind, parallel-group, active-controlled non-inferiority trials comparing oral daily vadadustat to parenteral DA in NDD-CKD patients, already treated for anemia and non-previously treated (naïve), respectively. Vadadustat did not meet the pre-specified non-inferiority criterion compared to DA with regards to cardiovascular safety. Interestingly, cardiovascular safety was similar between the two arms of treatment in the regions were the Hb target was 10–11 g/dL, but the cardiovascular risk was higher in patients randomized to vadadustat in regions with a Hb target of 10–12 g/dL.

Daprodustat has also been approved recently for use in Japan, and various phase III clinical trials are currently ongoing: ASCEND-D (NCT02879305), ASCEND-ID (NCT03029208), ASCEND-TD (NCT03400033), ASCEND-ND (NCT02876835), and ASCENDNHQ (NCT03409107). Daprodustat has already demonstrated its efficacy in managing anemia of CKD both in DD and NDD-CKD patients in phase II studies.

Lastly, molidustat, which is structurally different to the other study drugs mentioned before, is currently being evaluated in small phase III studies (NCT03351166, NCT03543657, NCT03418168, NCT03350321, NCT03350347). Several phase II studies, which are part of the DIALOGUE program, compared molidustat with either placebo or ESA therapy in CKD patients, demonstrating the efficacy, safety and tolerability of the drug (120).

By the time of elaboration of the present manuscript, the Cochrane Kidney and Transplant Group

Has published the protocol for a systematic review on HIF-PHIs for the treatment of anemia of chronic kidney disease (121).

### Safety of HIF Prolyl Hydroxylase Inhibitors

From a mechanistic point of view, the inhibition of prolyl-hydroxylases prevents HIF from degradation, leading to an increase of endogenous EPO within the physiological range, rather than the pharmacological levels achieved by current ESAs. Therefore, the rate of adverse events related to the high EPO levels should be expected to be lower than with ESAs. However, as mentioned before, HIF also modulates many other non-erythropoietic genes. This activity would explain the potential beneficial effects seen in pre-clinical and early clinical studies such as an improved iron utilization, HDL and LDL lowering effect, ischemia protection and a protective effect on CKD progression, improved neo-vascularization or better blood pressure control (122).

Nonetheless, potential deleterious side effects due to the modulation of other genes with this new class of drugs have also been postulated, notably tumor progression, enhanced vascular calcification, enhanced growth of renal cysts, worsening of retinopathy, or an increase in pulmonary artery pressure. In addition, whether these prolyl-hydroxylase inhibitors inhibit other di-oxygenases beyond HIF-PHIs and thus other pathways is unknown. Data from large phase III studies are still to be published and will surely help to answer these open questions.

In conclusion, in light of this evidence, HIF-PHIs, can be a viable alternative for the treatment of NDD and DD anemic CKD patients. However, more data is required regarding their long-term safety and their possible non-erythropoietic effects. In addition, the subset of patients that may benefit from these new agents still needs to be elucidated.

## Anemia Management, ESAs and COVID 19

Infection with severe acute respiratory syndrome coronavirus 2 (SARS-COV-2) has become a worldwide pandemic during 2020 and millions of cases have been reported worldwide. The coronavirus disease can lead to sepsis, acute kidney injury (AKI), multiple organ dysfunction and an atypical form of the acute distress respiratory syndrome. Anemia and a disturbed iron metabolism are common in COVID 19 patients. In an observational study Among 11,265 patients across 13 New York hospitals admitted between March 1 and April 27 2020, an elevation in D-dimer level was associated with a lesser median hemoglobin level and a greater serum ferritin level. And so are they in patients with COVID 19 suffering an AKI and in maintenance dialysis patients (123, 124). The exact mechanisms of COVID 19 are not completely known, but patients with a severe COVID 19 often present with an intense inflammatory phase and with a prothrombotic state. In these cases, the efficacy of ESAs is limited and they could even be potentially harmful. Fishbane et al. in a recent editorial article suggest avoiding ESA therapy (124). In the case of maintenance dialysis if the patient was already on that treatment, the authors recommend the continuation of ESA at the same dose but targeting a lower Hb targets (Hb 8–9 g/dL). Some other authors speculate on the potential role of HIF –PHDs as a protective agents against COVID. Indeed, the activation of the HIF1α pathway would decrease angiotensin convertase enzyme 2 (ACE2), which is the bound for COVID 19 membrane spike protein to enter the host cell, and therefore, decrease the invasiveness of SARS-CoV-2 (125).

Regarding iron supplementation, systemic inflammatory processes as happens with severe COVID 19 decrease the availability of iron. Furthermore, iron is also essential for viral replication (126). In addition, patients with viral infections and iron overload have a poor prognosis. Therefore, limiting iron supplements could be beneficial for patients with severe COVID 19 although more studies are need to shed light into this subject (127).

### Anemia in CKD-Key Points

- The pathophysiology of CKD-anemia is multifactorial, thus requiring a holistic approach
- Not all ESA are equal and whether their different pharmacokinetics and pharmacodynamics is associated with different outcomes in CKD patients remains to be elucidated.
- Iron is essential for other physiologic process beyond erythropoiesis. Observational studies in NDD-CKD patients suggest that iron deficiency is associated with worse outcomes, paving the way to randomized controlled trials that demonstrate the benefit of correcting iron deficiency beyond anemia.
- The upper limits of ferritin and TSAT indicating iron overload and risk of developing adverse events are still not clear, especially in the long-term.
- HIF prolyl hydroxylase inhibitors are new drugs under clinical evaluation. The available data suggest that they are efficacious and safe alternatives to ESA for the treatment of anemia in NDD and DD-CKD patients with several potential advantages over current therapies. However, more data is required to confirm these findings.

## Author Contributions

All authors contributed to the writing article and approved the submitted version.

## Conflict of Interest

The authors declare that the research was conducted in the absence of any commercial or financial relationships that could be construed as a potential conflict of interest.
